# Modified Framingham stroke risk profile and related research on uric acid in individuals aged over 65 years in the Yangtze River Delta region, China

**DOI:** 10.3389/fneur.2026.1773084

**Published:** 2026-03-18

**Authors:** Jing Yang, Jiasheng Zhang, Zhiqiang Qi

**Affiliations:** 1 Neurology Department, The Affiliated Jiangsu Shengze Hospital of Nanjing Medical University, Suzhou, China; 2 Health Monitoring Department, Suzhou High-tech Zone Center for Disease Control and Prevention, Suzhou, China

**Keywords:** community population, elderly population, modified Framingham stroke profile, stroke, uric acid

## Abstract

**Background:**

With socioeconomic advancement stroke has garnered extensive public attention.

**Aims:**

This study aimed to assess the 10-year risk of stroke among individuals aged over 65 years within a community utilizing the modified Framingham Stroke Profile (FSP). Additionally, the characteristics of high-risk factors for stroke and their associations with peripheral blood uric acid levels (UA) were explored.

**Methods:**

A cross-sectional stratified survey method was employed, adhering to the principles of stratified sampling. Between February and October 2020, residents from seven community committees in the Suzhou High-tech Zone were selected to participate in questionnaire surveys and undergo blood biomarker testing to evaluate the 10-year stroke risk of stroke using the modified FSP.

**Results:**

According to the population-based modified FSP risk assessment, 823 individuals (51.0%) were classified as low risk, 703 individuals (43.6%) as medium risk, and 87 individuals (5.4%) as high risk. Peripheral blood uric acid levels were positively correlated with the 10-year stroke risk score (*r* = 0.135). Logistic regression analysis identified several risk factors for modified FSP medium-high risk in the community-dwelling population aged over 65 years, comprising male sex, age, hyperlipidemia, and hyperuricemia in Q4. Moreover, receiver operating characteristic (ROC) curve analysis identified peripheral blood uric acid level as a potential predictor of high stroke risk, with an area under the ROC curve (AUC) of 0.611 (medium+high vs. low). Lastly, the optimal diagnostic threshold was 292.5 μmol/L (approximately 4.92 mg/dL).

**Conclusion:**

The stroke risk profile in this region warrants serious consideration. Elevated levels of uric acid (UA) are associated with increased modified FSP risk. Consequently, peripheral uric acid may serve as a limited indicator for discriminating medium-to-high modified FSP risk.

## 1 Introduction

With socioeconomic advancement improvements in living standards, changes in lifestyles patterns and ecological environments, and progressive population aging, stroke has garnered widespread public attention. As is well documented, stroke is characterized by the “five highs”: high incidence rate, high disability rate, high mortality rate, high recurrence rate, and high economic burden, making it the leading threat to public health. According to the Global Burden of Disease (GBD) study on stroke from 1990 to 2019 (1), approximately 12.2 million new cases of stroke were reported worldwide in 2019, with ischemic strokes accounting for 62.4% of cases. The total number of individuals affected by stroke was approximately 101 million. Moreover, stroke resulted in 143 million deaths and disability-adjusted life years (DALYs), with approximately 6.55 million deaths ascribed to the condition. By 2019, stroke had become the second leading cause of death globally, representing roughly 11.6% of all deaths. From 1990 to 2019, the incidence of new strokes increased by 70%, the total number of stroke patients rose by 85%, stroke-related deaths increased by 43%, and DALYs attributable to stroke grew by 32%. Despite being a severe and irreversible disease, stroke is also preventable and manageable. Consequently, early identification and screening are crucial for its prevention. Serum uric acid (UA), the end product of purine metabolism in nucleic acids, has been established in earlier studies to be associated with the development of various diseases, including hypertension, coronary heart disease, and stroke. At present, numerous domestic reports on population-based cardiovascular risk prediction have been published, and relevant predictive models have reached a relatively advanced stage of development (2–4). However, studies focusing on population-based stroke risk assessment remain scarce. Thus, this study employed the modified FSP to evaluate the risk of stroke in a specific population in the Suzhou High-tech Zone. Specifically, a survey was conducted among community-dwelling individuals aged 65 years and older to assess their 10-year stroke risk and to explore the relationship between modified FSP risk and uric acid levels. The objective of the present study was to provide evidence to inform the development of targeted interventions and preventive strategies for stroke among the elderly population in the community.

## 2 Participants and methods

### 2.1 Study population

This study employed a cross-sectional survey method using stratified sampling. From February to October 2020, elderly individuals aged over 65 years were selected from seven community neighborhood committees in a subdistrict of Suzhou High-tech Zone to participate in questionnaire surveys and blood biomarker testing. A total of 1,613 subjects were enrolled in the study, comprising 907 females (56.2%) and 706 males (43.8%), with ages ranging from 65 to 84 years and an average age of 76.76 ± 4.77 years. Uric acid levels were categorized into four quartiles: Q1 ≤ 259 μmol/L (403 cases), 260 μmol/L ≤ Q2 ≤ 308 μmol/L (403 cases), 309 μmol/L ≤ Q3 ≤ 370 μmol/L (404 cases), and Q4 ≥ 371 μmol/L (400 cases). Three individuals had complete baseline data but lacked uric acid measurements. All studies involving human subjects were reviewed by the Ethics Committee of Jiangsu Shengze Hospital and conducted in accordance with the principles of the Declaration of Helsinki (Ethics No: 2020-004-01). Informed consent was obtained from all participants prior to study enrollment.

### 2.2 Eligibility criteria

**The inclusion criteria for this study were as follows:** ① Residing on a specific street in the Suzhou High-tech Zone for 6 months or longer; ② Aged 65 years or older.

**Exclusion Criteria:** ① History of stroke; ② Diagnosis of malignant tumors; ③ Presence of psychiatric disorders or unwillingness to cooperate with the investigation.

The exclusions for “history of stroke” and “malignant tumors” are based on self-reports and reviews of medical records.

### 2.3 Data collection

Demographic and clinical data were collected from the elderly population in the community, encompassing sex, age, body mass index (BMI), family history of stroke, and physical inactivity level. Additionally, fasting venous blood samples were obtained to assess serum cholesterol (total cholesterol, TC), triglyceride (TG), low-density lipoprotein cholesterol (LDL-C), high-density lipoprotein cholesterol (HDL-C), and uric acid (UA) levels.

### 2.4 Modified FSP assessment for stroke risk

The modified FSP incorporates various risk factors, including age, systolic blood pressure, systolic blood pressure after antihypertensive treatment, history of diabetes, smoking status, history of cardiovascular disease, history of atrial fibrillation, and history of left ventricular hypertrophy, to develop a mathematical model that predicts the 10-year incidence of stroke. Higher assessment scores correspond to an increased risk of stroke within this period. For males, a score of 21 to 30 indicates high risk, 11 to 20 signifies medium risk, and 1 to 10 reflects low risk. For females, scores of 19 to 27 are categorized as high risk, 10 to 18 as medium risk, and 1 to 9 as low risk (5).

### 2.5 Statistical analysis

Statistical analyses were conducted using SPSS 22.0 statistical software. Normally distributed data were expressed as (Mean ± standard deviation), and categorical data were compared using the chi-squared (*x*^2^) test. UA levels were categorized into quartiles, with the Q1 group serving as the reference category. Logistic regression analysis was carried out to enhance the multifactorial analysis of medium-high risk factors associated with modified FSP risk. Additionally, ROC curve analysis was conducted to evaluate the predictive capability of peripheral blood UA levels for identifying patients at medium-high risk of modified FSP. *P* < 0.05 was considered statistically significant.

## 3 Results

### 3.1 Exposure to risk factors among the survey subjects

Among the 1,613 elderly individuals aged 65 years and older, 907 were female (56.2%), and 706 were male (43.8%). Among them, 603 (37.4%) were aged between 65 and 74 years, whilst the remaining 1,010 individuals were aged between 75 and 84 years. A total of 615 individuals (38.1%) were classified as overweight based on Body Mass Index (BMI), whilst 184 individuals (11.4%) were categorized as obese. Besides, 834 participants (51.7%) reported being physically inactive, 748 individuals (46.4%) suffered from hyperlipidemia, and 26 participants (1.6%) reported a family history of stroke.

### 3.2 Comparison of data across modified FSP-based risk categories in individuals aged over 65 years

According to the modified FSP assessment for individuals aged over 65 years, 823 participants (51.0%) were classified as low risk, 703 participants (43.6%) as medium risk, and 87 participants (5.4%) as high risk. Notably, the proportion of males categorized as high risk (70.1%) was significantly greater than that of women. Furthermore, significant differences were noted among various risk groups, namely sex, age, triglyceride (TG) levels, high-density lipoprotein cholesterol (HDL-C) levels, presence of hyperlipidemia, and uric acid levels (*P* < 0.05). Additionally, with increasing modified Framingham risk levels, a corresponding rise in the proportion of individuals in the fourth quartile (Q4) of high uric acid levels was observed, as evidenced by the chi-squared test for trend (*P* < 0.05). Detailed results are listed in Table 1.

**Table 1:** Comparison of data across modified FSP-based risk categories in individuals aged over 65 Years.

**Characteristics**	**Low risk (823, 51.0%)**	**Medium risk (703, 43.6%)**	**High risk (87, 5.4%)**	***F*/*x*^2^**	***P***
**Sex**					
Male (*n*, %)	293 (35.6%)	352 (50.1%)	61 (70.1%)	58.187	0.000^a^
Female (*n*, %)	530 (64.4%)	351 (49.9%)	26 (29.9%)		
Age ($\bar{x}$ *± s*)	74.71 ± 4.15	79.46 ± 4.17^b^	74.28 ± 3.86^c^	264.913	0.000^a^
BMI ($\bar{x}$ *± s*)	24.04 ± 3.26	23.99 ± 3.29	24.72 ± 2.96	1.961	0.141
TC ($\bar{x}$ *± s*)	4.61 ± 0.99	4.67 ± 0.95	4.79 ± 1.46	1.615	0.199
TG ($\bar{x}$ *± s*)	1.54 ± 1.41	1.49 ± 0.85^b^	1.95 ± 2.05^c^	5.330	0.005^a^
LDL-C ($\bar{x}$ *± s*)	2.82 ± 0.80	2.90 ± 0.83	2.90 ± 1.22	1.777	0.169
HDL-C ($\bar{x}$ *± s*)	1.32 ± 0.31	1.29 ± 0.33^b^	1.21 ± 0.32^c^	5.743	0.003^a^
Hyperlipidemia (*n*, %)	299 (36.3%)	394 (56.0%)	55 (63.2%)	69.751	0.000^a^
Family history of stroke (*n*, %)	13 (1.6%)	11 (1.6%)	2 (2.3%)	0.274	0.872
Lack of exercise (*n*, %)	439 (53.6%)	360 (51.4%)	35 (41.2%)	4.947	0.084
**Uric acid quartile**					
Q1 (*n*, %)	261 (31.7%)	126 (18.0%)	16 (18.4%)	62.933	0.000^a^
Q2 (*n*, %)	213 (25.9%)	179 (25.6%)	11 (12.6%)		
Q3 (*n*, %)	185 (22.5%)	196 (28.0%)	23 (26.4%)		
Q4 (*n*, %)	164 (19.9%)	199 (28.4%)	37 (42.5%)		

^a^indicates *P* < 0.05, ^b^indicates a significant difference compared to the low-risk group, and ^c^ signifies a significant difference compared to the medium-risk group.

### 3.3 Correlation between peripheral uric acid levels and modified FSP risk in individuals over 65 years old

Pearson correlation analysis indicated that peripheral blood uric acid levels were positively correlated with the 10-year stroke risk score (*r* = 0.135, *P* = 0.000). In males, peripheral blood uric acid levels were positively correlated with the 10-year stroke risk score (*r* = 0.053, *P* = 0.157). Likewise, a positive correlation was observed between peripheral blood uric acid levels and 10-year stroke risk score in females (*r* = 0.139, *P* = 0.000).

### 3.4 Multivariate analysis of medium-high risk modified FSP factors in individuals aged 65 and above in the community

Based on the modified FSP assessment, comparisons were conducted between the low-risk group and the medium-high-risk group. Due to the collinearity among lipid indicators, a multifactorial logistic regression analysis was conducted using sex (0 = female, 1 = male), age, BMI, hyperlipidemia, and uric acid levels as independent variables. The results identified the following risk factors for modified FSP medium-high risk among elderly individuals over 65 years in the community: male sex (*OR* = 1.897, *P* = 0.000), age (*OR* = 1.231, *P* = 0.000), hyperlipidemia (*OR* = 2.535, *P* = 0.000), and uric acid levels, with odds ratios of 1.412, 1.542, and 1.541 for Q2, Q3, and Q4, respectively (*P* < 0.05 for all), detailed in Table 2.

**Table 2:** Multivariate analysis of medium-high risk factors for modified FSP in individuals aged 65 and above in the community.

**Variables**	***B***	***Wald***	***OR***	***95%CI***	***P***
Male	0.640	25.885	1.897	1.482–2.428	0.000^a^
Age	0.208	253.295	1.231	1.200–1.263	0.000^a^
BMI	−0.027	2.028	0.974	0.939–1.010	0.154
Hyperlipidemia	0.930	58.031	2.535	1.995–3.220	0.000^a^
Uric acid (with Q1 as reference)					0.049^a^
Q2	0.345	4.304	1.412	1.019–1.955	0.038^a^
Q3	0.433	6.258	1.542	1.098–2.165	0.012^a^
Q4	0.432	5.658	1.541	1.079–2.200	0.017^a^

^a^indicates *P* < 0.05.

### 3.5 Prediction of modified FSP medium-high risk by peripheral blood uric acid in community-based elderly individuals aged over 65 years

**Figure 1:**
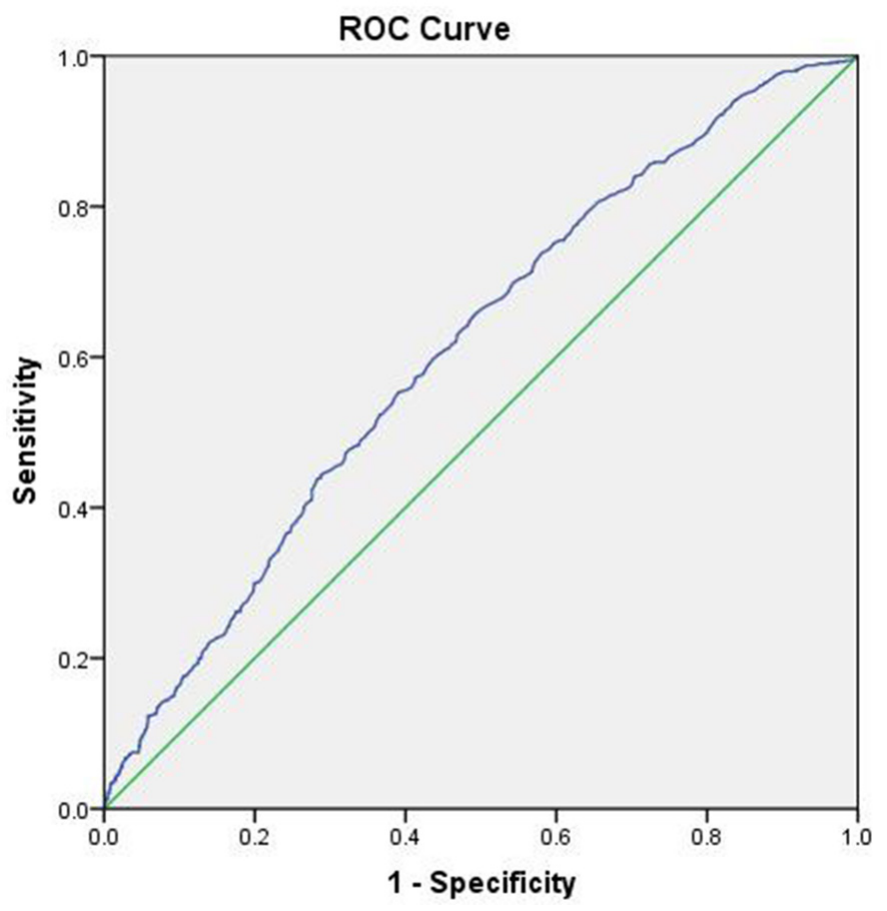
ROC curve for predicting modified FSP medium-high risk by peripheral blood uric acid in community-based elderly individuals aged over 65 years.

The predictive value of peripheral blood uric acid for identifying high stroke risk in individuals aged over 65 years was assessed using ROC curve analysis. The AUC was 0.611 (medium+high vs. low). More importantly, the optimal diagnostic threshold was established at 292.5 μmol/L (approximately 4.92 mg/dL), with a sensitivity of 66.5% and a specificity of 50.2% (see Figure 1).

## 4 Discussion

Stroke, an acute cerebrovascular condition, poses a significant threat to public health. A study examining the burden of stroke in China revealed that in 2019 (6), there were approximately 3.94 million new stroke cases, affecting a total of 28.76 million individuals (24.18 million with ischemic stroke, 4.36 million with intracerebral hemorrhage, and 1.58 million with subarachnoid hemorrhage). Moreover, stroke-related mortality reached 2.19 million, while the number of Disability-Adjusted Life Years (DALYs) lost was 45.9 million. Compared to 1990, the incidence rate increased by 86.0%, the prevalence rate rose by 106.0%, the mortality rate grew by 32.3%, and the DALY rate decreased by 41.6%. While advancements in stroke prediction, treatment, and rehabilitation have been made, primary prevention remains the most cost-effective strategy for reducing the burden of stroke (7, 8). Risk prediction serves as an effective tool to identify high-risk populations at high risk of both stroke occurrence and recurrence, thereby informing preventive strategies. This holds significant implications for stroke prevention. To date, several well-established risk assessment tools for ischemic stroke are available both domestically and internationally, including the modified FSP, Pooled Cohort Equations, and Stroke Riskometer, which are utilized for assessing the risk of ischemic stroke and guiding preventive strategies (9–12).

The modified FSP originated from D'Agostino et al.'s 1994 enhancement of the original FSP. The revised FSP has been endorsed by several authoritative guidelines, including the 2011 AHA/ASA Primary Prevention of Stroke Guidelines, and has received recognition from the Chinese Neurological Society as a risk assessment tool for primary prevention. This screening scale is straightforward and cost-effective, making it appropriate for community screening and primary healthcare applications (13). To date, studies focused on improving the predictive ability of FSP remain limited. Onwuakagba et al. (14) indicated that the 10-year predicted risk of stroke in the community population was 84%, 10%, and 6% for low-, medium-, and high-risk patients, respectively. In the present modified FSP assessment of the elderly population aged 65 years and above, 823 individuals (51.0%) were classified as low risk, 703 individuals (43.6%) as medium risk, and 87 individuals (5.4%) as high risk. Compared to the aforementioned reports, the proportion of individuals at risk of stroke in this study population appears to be relatively high. This discrepancy may be attributed to the characteristics of research subjects. Specifically, patients recruited in this study were elderly individuals aged 65 years and above who may possess additional risk factors and multiple comorbidities, leading to a higher proportion of individuals categorized as medium risk. Of note, this survey revealed that the proportion of males at high risk for stroke was higher, at 70.1%. Male sex (*OR* = 1.897) was identified as a significant risk factor for medium-high modified FSP risk, in line with the observation of Xing et al. (15), which may be ascribed to the higher rates of smoking preference for high-fat foods, and unhealthy lifestyle choices among males. Additionally, Wang et al. (16) documented that the age-specific incidence of total stroke and its three primary pathological types (ischemic stroke, intracerebral hemorrhage, and subarachnoid hemorrhage) increased with age (*P* < 0.05), in line with the results of this survey. Noteworthily, comparing the stroke groups unveiled significant differences in age (*P* < 0.05) (*OR* = 1.231), which was identified as a significant risk factor for medium-high modified FSP risk, supporting the established association between increasing age and elevated stroke risk. Furthermore, the results of this study identified dyslipidemia (*OR* = 2.535) as a significant risk factor for medium-high modified FSP risk, implying that abnormal blood lipid levels contribute to the development of atherosclerosis. High blood lipid levels may compromise the stability of blood vessel walls, thereby increasing the risk of hemorrhagic events (17–19).

Previous research has demonstrated that uric acid exerts a dual influence on cerebrovascular diseases. As a renewable antioxidant, it possesses a robust capacity to resist apoptosis, playing a key role in maintaining the body's immune response and mitigating neural damage associated with ischemic cerebrovascular diseases. At the same time, its potent antioxidant properties protect neurons from oxidative stress-induced damage and alleviate ischemic injury by inhibiting lipid peroxidation. Furthermore, uric acid preserves the function of vascular endothelial cells by preventing the degradation of extracellular superoxide dismutase (SOD3), a vital enzyme in sustaining endothelial and vascular health (20–22). Conversely, under certain conditions, uric acid can act as a pro-oxidant, exacerbating atherosclerosis formation by promoting the oxidation of low-density lipoprotein cholesterol and lipid peroxidation reactions. Simultaneously, it can also activate platelet function, leading to platelet adhesion, initiation of the coagulation cascade, and increased thrombotic risk, Besides, uric acid drives vascular endothelial cell dysfunction, promotes the generation of local oxidants, and triggers inflammatory mediator responses, thereby aggravating ischemic hypoxia and brain tissue damage (23, 24). Zhong et al. (20) analyzed 13 prospective studies and concluded that for every 1 mg/dL increase in serum uric acid levels, the relative risk (*95% CI*) of stroke was 1.10 (1.05–1.14) in males and 1.11 (1.09–1.13) in females, with no statistically significant difference in the future risk of stroke (*P* = 0.736). At the same time, Meanwhile, Yang et al. (25) recruited young patients and described that, compared to the mild stroke group, individuals in the severe stroke group were generally older and exhibited higher blood glucose levels, systolic blood pressure, serum triglyceride levels, low-density lipoprotein cholesterol levels, and uric acid (SUA) levels (*P* < 0.05). It is worthwhile emphasizing that compared to the moderate stroke group, the severe stroke group had a higher proportion of males, older individuals, smokers, and individuals with a family history of stroke, diabetes, and cardiovascular disease (*P* < 0.05). Multivariate logistic regression analysis revealed a significant association between blood SUA levels and stroke risk. Zheng et al. (26) conducted a seven-year prospective cohort study in Northwest China, involving a total of 23,262 individuals without cardiovascular disease from the Jinchang cohort. These participants were followed for an average duration of 5.26 years. As anticipated, baseline serum uric acid (SUA) levels and their relative changes were positively correlated with the incidence of stroke in both males and females (*p* for overall association < 0.0001). In addition, the risk of stroke increased by 4.6% for every 10% increase in the relative SUA changes (*HR* = 1.046, 95% CI, 1.007–1.086). Fully adjusted regression analysis revealed that only substantial increases in SUA (>30%) were associated with a 36.5% increased risk of stroke, compared to the reference group (participants with ± 10% changes in SUA). This trend was also observed in individuals with normal baseline SUA levels. A retrospective, single-center observational study conducted by Sengüldür and Demir (27) involving 31,202 patients presenting to the emergency department demonstrated that stroke patients exhibited significantly higher median serum uric acid (SUA) levels (*P* < 0.001). Furthermore, hyperuricemia (defined as UA ≥ 7 mg/dL) was associated with a 2.4-fold increased risk of ischemic stroke (odds ratio: 2.402, *P* < 0.001). In contrast, hypouricemia (defined as UA ≤ 2.8 mg/dL) was linked to a reduced risk of ischemic stroke (odds ratio: 0.272, *P* < 0.001). These findings collectively suggest that serum uric acid (SUA) levels are associated with ischemic stroke and that hyperuricemia can serve as an adjunctive parameter to enhance the diagnosis of stroke in the emergency department (ED). The results of this study corroborate the findings of previous reports, given the significant differences observed across different stroke and uric acid groups (*P* < 0.05). Furthermore, an increasing modified Stroke Framingham Profile risk level was associated with a higher proportion of individuals in Q4 (high-level uric acid) (χ^2^ trend test, *P* < 0.05). Peripheral blood uric acid levels were positively correlated with the 10-year stroke risk score (*r* = 0.135). Notably, uric acid (Q4 *OR* = 1.541) was identified as a risk factor for modified FSP medium-high risk in community-dwelling individuals aged over 65 years. Mounting evidence suggests that individuals with high serum uric acid levels may face a greater risk of future stroke compared to those with low serum uric acid levels. Further receiver operating characteristic (ROC) curve analysis displayed that serum uric acid levels in peripheral blood could predict a heightened risk of stroke, with an area under the ROC curve (AUC) of 0.611 (medium+high vs. low) and an optimal diagnostic threshold of 292.5 μmol/L. Nevertheless, this cut-point is derived from the dataset and necessitates external validation prior to clinical adoption. Serum uric acid levels are influenced by renal function, diuretics, urate-lowering therapy, diet, and alcohol consumption, as well as metabolic comorbidities. Moreover, some limitations of this study merit acknowledgment. To begin, this study did not include renal function parameters (such as creatinine or eGFR) and related medications (diuretics and urate-lowering agents) in the baseline characteristics and sensitivity analyses. Elevated uric acid (UA) levels promote the proliferation of vascular endothelial cells, resulting in endothelial dysfunction and increased expression of pro-inflammatory mediators in vascular smooth muscle cells. Additionally, UA accelerates lipid peroxidation, facilitates low-density lipoprotein (LDL) oxidation, and inhibits nitric oxide (NO) synthesis, all of which contribute to vascular endothelial dysfunction. High UA levels also enhance the production of platelet-derived growth factor, which promotes platelet adhesion and activates the coagulation cascade, ultimately leading to thrombosis and arterial occlusion. Furthermore, elevated UA levels can increase the levels of systemic inflammatory factors and induce systemic inflammatory responses via the NF-κB pathway. The production of UA is accompanied by the generation of reactive oxygen species, which can induce oxidative stress and lead to endothelial cell apoptosis. Finally, UA can activate the renin-angiotensin-aldosterone system (RAAS), thereby increasing renin activity, promoting angiotensin II synthesis, and enhancing water and sodium retention, which collectively increase vascular resistance and the risk of ischemic events (28–30).

This study acknowledges several limitations. First, our research aimed to concentrate on a specific aging population residing in urban communities within economically developed regions. The elderly population in a particular subdistrict of Suzhou New District, as a representative area of the Yangtze River Delta, displays distinctive characteristics regarding health status, medical accessibility, and lifestyle. These findings may offer valuable insights into issues concerning the elderly population in urban communities of developed regions. However, caution should be exercised when generalizing the results of this study to other regions of China, particularly rural or less developed areas, younger populations, or individuals from diverse socioeconomic backgrounds.

Another limitation is that although the modified FSP has been validated in Western populations, domestic validation indicates that while it effectively predicts stroke risk (AUC: 0.82 for males, 0.82 for females) (13), it significantly underestimates the absolute risk of stroke. In future research, we will consider employing more population-specific tools, such as the China-PAR model.

Future research should employ a multicenter, prospective design that facilitates cross-regional collaboration. This approach should incorporate a more diverse and representative sample population to further validate and refine our conclusions.

## 5 Conclusions

In summary, the modified FSP risk assessment conducted among the elderly population aged 65 years and above in this community uncovered that 823 individuals (51.0%) were classified as low risk, 703 individuals (43.6%) as medium risk, and 87 individuals (5.4%) as high risk. These findings highlight the necessity of addressing stroke risk status within this population. Identified risk factors for elevated medium-high modified FSP risk in the elderly included male sex, age, hyperlipidemia, and hyperuricemia (UA). Notably, elevated levels of uric acid (UA) were associated with higher modified FSP risk. Overall, these findings position blood uric acid levels as a limited indicator for discriminating individuals at medium-to-high modified FSP risk. Early screening strategies, informed by population characteristics, and targeted interventions are recommended to minimize the incidence of cerebrovascular diseases.

## Data Availability

The raw data supporting the conclusions of this article will be made available by the authors, without undue reservation.
